# Dissecting Sex-Related Cognition between Alzheimer's Disease and Diabetes: From Molecular Mechanisms to Potential Therapeutic Strategies

**DOI:** 10.1155/2021/4572471

**Published:** 2021-03-05

**Authors:** Ghulam Md Ashraf, Mahmoud Ahmed Ebada, Mohd Suhail, Ashraf Ali, Md. Sahab Uddin, Anwar L. Bilgrami, Asma Perveen, Amjad Husain, Mohd Tarique, Abdul Hafeez, Athanasios Alexiou, Ausaf Ahmad, Rajnish Kumar, Naheed Banu, Agnieszka Najda, Amany A. Sayed, Ghadeer M. Albadrani, Mohamed M. Abdel-Daim, Ilaria Peluso, George E. Barreto

**Affiliations:** ^1^King Fahd Medical Research Center, King Abdulaziz University, Jeddah, Saudi Arabia; ^2^Department of Medical Laboratory Technology, Faculty of Applied Medical Sciences, King Abdulaziz University, Jeddah, Saudi Arabia; ^3^Faculty of Medicine, Zagazig University, Zagazig, El-Sharkia, Egypt; ^4^National Hepatology and Tropical Medicine Research Institute, Cairo, Egypt; ^5^Department of Sciences of Agriculture, Food, Natural Resources, and Engineering (DAFNE), University of Foggia, Via Napoli 25, 71122 Foggia, Italy; ^6^Department of Pharmacy, Southeast University, Dhaka, Bangladesh; ^7^Pharmakon Neuroscience Research Network, Dhaka, Bangladesh; ^8^Department of Entomology, Rutgers University, New Brunswick, NJ 018901, USA; ^9^Deanship of Scientific Research, King Abdulaziz University, Jeddah, Saudi Arabia; ^10^Glocal School of Life Sciences, Glocal University, Saharanpur, India; ^11^Centre for Science and Society, IISER Bhopal, India; ^12^Innovation and Incubation Centre for Entrepreneurship, IISER Bhopal, India; ^13^Department of Child Health, University of Missouri, Columbia, MO 65201, USA; ^14^Glocal School of Pharmacy, Glocal University, Saharanpur, India; ^15^Novel Global Community Educational Foundation, New South Wales, Australia; ^16^AFNP Med Austria, Wien, Austria; ^17^Amity Institute of Biotechnology, Amity University Uttar Pradesh Lucknow Campus, Uttar Pradesh, India; ^18^Department of Physical Therapy, College of Medical Rehabilitation, Qassim University, Buraidah, Qassim, Saudi Arabia; ^19^Laboratory of Quality of Vegetables and Medicinal Plants, Department of Vegetable Crops and Medicinal Plants, University of Life Sciences in Lublin, 15 Akademicka Street, 20-950 Lublin, Poland; ^20^Zoology Department, Faculty of Science, Cairo University, Giza 12613, Egypt; ^21^Department of Biology, College of Science, Princess Nourah bint Abdulrahman University, Riyadh 11474, Saudi Arabia; ^22^Pharmacology Department, Faculty of Veterinary Medicine, Suez Canal University, Ismailia 41522, Egypt; ^23^Research Centre for Food and Nutrition, Council for Agricultural Research and Economics (CREA-AN), 00142 Rome, Italy; ^24^Department of Biological Sciences, University of Limerick, Limerick, Ireland; ^25^Health Research Institute, University of Limerick, Limerick, Ireland

## Abstract

The brain is a sexually dimorphic organ that implies different functions and structures depending on sex. Current pharmacological approaches against different neurological diseases act distinctly in male and female brains. In all neurodegenerative diseases, including Alzheimer's disease (AD), sex-related outcomes regarding pathogenesis, prevalence, and response to treatments indicate that sex differences are important for precise diagnosis and therapeutic strategy. Pathogenesis of AD includes vascular dementia, and in most cases, this is accompanied by metabolic complications with similar features as those assembled in diabetes. This review discusses how AD-associated dementia and diabetes affect cognition in relation to sex difference, as both diseases share similar pathological mechanisms. We highlight potential protective strategies to mitigate amyloid-beta (A*β*) pathogenesis, emphasizing how these drugs act in the male and female brains.

## 1. Introduction

Alzheimer's disease (AD) is the most common dementia-related disorder, which has shown an alarming rise in its prevalence around the world, and its number is expected to rise over a hundred million by 2050 [1, 2]. Emerging evidence indicates that the pathogenesis of AD is attributable to chronic vascular pathologies [3]. Thus, AD may be considered a vascular disorder with neurodegenerative consequences rather than being a neurodegenerative disorder. Biochemically, AD is characterized by amyloid-beta (A*β*) plaque formation and tau protein hyperphosphorylation inside neurons [4, 5]. The dominant hypothesis regarding AD (i.e., the amyloid cascade hypothesis) suggests that increased accumulation of A*β* peptide in the brain parenchyma leads to memory loss and cognitive decline that are clinical characteristics of the disease [6]. The conventional hypothesis is that AD precedes vascular dysfunction. Elevated cytokine expression and microglial activation are also contributors to neuroinflammatory changes in AD [7]. Clinically, the disease worsens by memory impairment and a decline in cognitive ability, leading to eventual death [8]. Effective therapies for AD have not yielded significant outcomes so far. However, the social and economic cost of caring for AD patients makes it rational to continue searching for effective therapies.

Important key factors that play critical roles in AD pathogenesis include aging and decreased cerebral perfusion. With advancing age, cerebral blood flow decreases to lower the brain perfusion, thus placing vulnerable neurons in a state of high energy demand, consequently leading to a cascade of neuronal metabolic turmoil. A culmination of these two factors leads to the critically attained threshold of cerebral hypoperfusion. Cerebral hypoperfusion is a type of hemodynamic microcirculatory insufficiency, which can destabilize neurons, synapses, neurotransmission, and cognitive functions. These alterations can create a neurodegenerative condition with senile plaque formation, neurofibrillary tangles (NFTs), and amyloid angiopathy, leading to cognitive impairment [9, 10]. This alternative theory of AD pathogenesis has been supported by experimental studies, in which the blood-brain barrier (BBB) dysfunctions and impaired cerebral blood flow (CBF) have been observed [11].

Recent research findings indicate that pathological cerebral angiogenesis may occur due to A*β* accumulation, resulting in BBB dysfunction in AD. The abnormal increase of A*β* concentration in blood circulation leads to decreased nitric oxide (NO) and vascular sensitivity to endothelium-dependent vasodilatation. This phenomenon can lead to constriction of blood vessels and ischemia in neighboring tissues [12]. Also, the increase of A*β* leads to cell death and decreased maximum vasodilator response of cerebral vessels in the context of AD [13].

**Figure 1:**
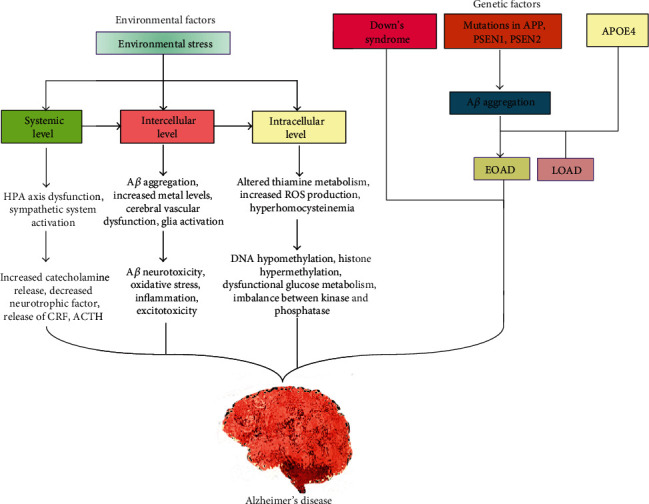
Genetic and environmental factors causing Alzheimer's disease.

Epidemiologic studies confirmed that genetic factors play an important role in the progression of early-onset AD (EOAD) and late-onset AD (LOAD). The mutations in amyloid precursor protein (*APP*), presenilin-1 (*PSEN1*), and presenilin-2 (*PSEN2*) are inherited in a Mendelian fashion, directly causing the EOAD, while genome-wide association studies (GWAS) have discovered many vulnerable genes influencing the susceptibility to LOAD [14]. AD-associated mutations in these three genes (i.e., *APP*, *PSEN1*, and *PSEN2*) show high penetrance (i.e., more than 85%), are generally autosomal dominantly inherited, and inevitably cause A*β* aggregation and EOAD (Figure 1).

On the other hand, LOAD-developing genes inherited in a non-Mendelian fashion raise the disease risk. First-degree relatives of patients with LOAD have two times predictable existence risk without an AD-affected first-degree relative. Additionally, LOAD appears to be more frequent in monozygotic than in dizygotic cotwins, which shows a major genetic contribution in the development of the disease [15]. The identification of the *ε*4 allele of apolipoprotein E (*APOE ε4*) is a well-established genetic risk factor for both EOAD and LOAD [16], which has unfolded new findings of at least 21 extra genetic risk loci for the genetically complex form of AD, emerging from GWAS and massively parallel resequencing efforts. These advances in AD genetics are positioned in light of the current endeavor directed towards translational research and personalized treatment of AD.

Even though genetic factors are inherited and fixed, other nongenetic factors contribute to the ailments. These factors include occupational exposures (i.e., pesticide spray, exposure to electromagnetic fields, volatile anesthetics, and organic solvents), lifestyle factors (i.e., alcohol consumption, smoking, drinking coffee, body mass index, cognitive activity, and physical activity), and preexisting diseases (i.e., traumatic brain injury, depression, hypertension, cerebrovascular disease, diabetes, dyslipidemia, and cancer) [17]. Copper ion can cause extended conformation of *Aβ*-peptides, which are correlated to AD [18]. Similarly, high levels of metals like aluminum (Al), zinc (Zn), and iron (Fe) in the brain can also lead to the generation of AD [19]. Lead can be absorbed by lung epithelial cells and the gastrointestinal tract upon binding to heme groups in the blood circulation. Environmental factors or insults such as environmental toxins, heat, starvation, psychological stress, hypothermia, glucose hypometabolism, anesthesia, brain trauma, and injury can stimulate hyperphosphorylation of tau protein, A*β* aggregation, and oligomerization, which are closely related to LOAD progression [20].

Diabetes mellitus (DM) is a complicated disorder that affects all tissues and organs, with metabolic complications reaching faraway to impaired glucose metabolism [21]. Type 1 diabetes mellitus (T1DM) is an autoimmune disorder, where the insulin-producing pancreatic *β*-cells are destroyed, preventing the body from producing a sufficient level of insulin hormone to control normal blood glucose levels. T1DM is mainly diagnosed in children and referred to as juvenile diabetes, which can develop at any age [22], while Type 2 diabetes mellitus (T2DM) is thought to occur in its earliest phase, from the declining sensitivity of peripheral tissues to circulating insulin. It may lead to impaired glucose tolerance (ITG), the proportional inadequacy of insulin, and compensatory hyperinsulinemia to manage glucose homeostasis [23].

The number of diabetic patients with cognitive impairment has been continuously increasing. Numerous epidemiological studies have linked DM with the occurrence of AD. Insulin resistance has been proposed as the mechanism by which DM increases AD-associated pathology [24–27]. The global economic burden of AD-dementia care and cure is enormous. Hence, there is an immediate need for sex-based studies to assess AD-associated dementia and therapeutic strategies [28–30]. This is an important consideration, especially in DM. In the present review, we outline the link between DM and AD and dissect sex-dependent common features on cognition between these two pathologies.

## 2. Sex Differences in Cognition for AD Patients

Sex differences regarding AD incidence are less clear. A Philadelphia Neurodevelopment Cohort study based on brain imaging between 8 and 22 years has explained developmental sex differences [31]. Previous literature has highlighted some scenarios that could affect AD dementia based on sex differences. These scenarios include (a) risk factors with the same frequency for both genders that have a more significant effect on one sex or another, such as *APOE* genotype as well as other genetic variants located on autosomal chromosomes; (b) risk factors with the same effect in both genders but with different frequencies, e.g., historically, males smoke more frequently, and females have less access to education; (c) risk factors with different frequencies and effects based on gender, e.g., males are more likely to have head traumas, while females are more prone to head injury's adverse events; and (d) risk factors that are limited to one sex, e.g., prostate cancer and androgen deprivation therapy in men as well as pregnancy and oophorectomy in women [32].

Evidence from many countries documented a higher incidence rate of AD dementia in old-aged women than men [33, 34]. This is supported by estimates, which indicate that almost two-thirds of patients diagnosed with AD are women. A recent meta-analysis among Asians, Europeans, and Americans has shown that women are at greater risk of developing AD-associated dementia than men [35]. There is a vague explanation for this difference, but it is assumed that the greater percentage of women among AD patients is due to their longer lifespan than the opposite sex [33]. Other studies conducted in Asian [36, 37] and European [38–41] populations showed similar trends. However, this estimate is not well supported across all the world regions. In the United States, numerous studies did not find sex-based differences in AD development [42–47]. Another recent study conducted by the Mayo Clinic showed that the rate of progression of mild cognitive impairment (MCI) to AD is identical in both sexes in the age group of 70-79 years, but it also reported cases of AD progression in women older than 80 years [48, 49]. Contrary to these findings, a recent report documented a higher incidence rate of AD dementia in men. Moreover, they mentioned a decline in dementia incidence in men (0.6; 95% CI: 0.4–0.9) but not in women (1.0; 95% CI: 0.7–1.3) [50]. Also, the Cache County Study (USA) reported a higher AD occurrence in men than women up to age 78. However, older subjects exhibited a reverse trend [51].

Until now, no exact reasons are established for the abovementioned discrepancy. However, time and geographic region may affect the incidence of AD dementia [52, 53]. In a recent longitudinal population-based study, neuropathic abnormalities were more common in white women, but microinfarcts were more common in Japanese-American men [54]. There may be variations in sample, size, and age distribution in addition to social, cultural, and historical factors in sex-based results in Asian, European, and American studies. Inflammation can be another cause of dementia in AD, where different effects have been observed in males and females. Inflammatory dysregulation is also found to be more prominent in females [55, 56]. There is a significant sex difference in microglia during development, which is a primary immune cell in the central nervous system. During the adolescent period, women have more microglia than men, when women-linked disorders such as depression and anxiety tend to rise. There is a possibility that disturbance in microglia at this developmental stage leads to neurodegenerative disorders in later stage of adulthood [55–57]. Low-grade inflammation is a risk factor for several medical implications like T2DM, obesity, anxiety, and depression. All such risk factors develop AD and other types of dementia in humans [58]. Thus, comprehensive global studies to assess the risk factors for AD dementia among both men and women are warranted to understand these differences.

## 3. Sex Hormones and Risk of AD

The fully grown brain structure and its development, as well as the brain activities and biochemistry, vary among genders [59]. Sex-determining genes and fetal hormonal programming trigger such differences in both male and female brains. These types of differences have imperative implications for brain-based disease risk and clinical and investigational approaches. Altmann et al. [60] documented that women who are positive for *APOE ε4* have a higher risk of having AD than men who are positive for this allele since they show more prevalent behavioral disinhibition [61].

**Figure 2:**
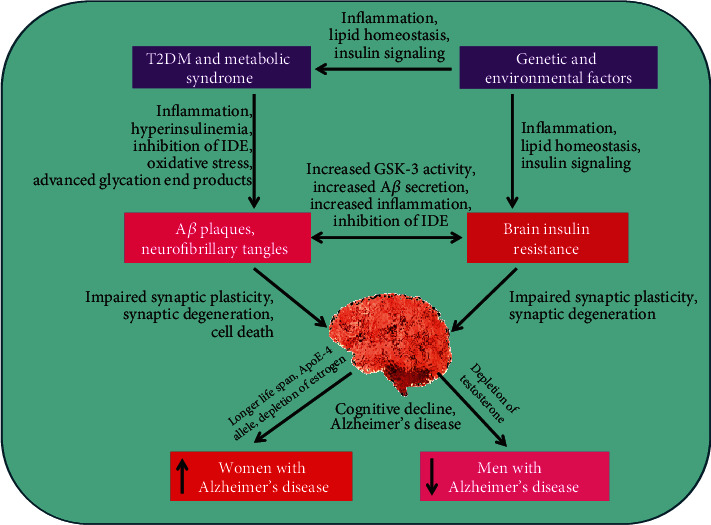
Sex differences with Alzheimer's disease and diabetes.

Some studies have unveiled that men with AD have different levels of sex hormones than normal men. Hence, male sex hormones have been hypothesized as AD-developing risk factors through immunomodulatory effects on known inflammatory AD risk factors, such as tumor necrosis factor-alpha (TNF-*α*) [62]. Barron and Pike [63] have demonstrated that age-related depletion of estrogen hormone in women and testosterone in men establishes risk factors for AD development. Generally, sex steroid hormones have anti-inflammatory activities, which may have interactions with many other AD-developing agents (Figure 2).

Progesterone plays an essential role in females during the childbearing period, but after menopause, there is a rapid decline in ovarian sex hormones such as 17 beta-estradiol and progesterone. Before menopause, oophorectomy leads to a significant loss of estrogen, progesterone, and testosterone, disrupting the hypothalamic-pituitary axis [64]. There is a reduction of male sex hormones with increasing age, but its impact is not as severe as in female sex hormones (e.g., progesterone and estrogen).

Several animal and cellular studies have proven the neuroprotective effect of estrogenic compounds [65–76]. Studies on various animal and cellular models have shown enhanced synapse formation on hippocampal dendritic spines, maintaining hippocampal function while aging [77–80]. When estrogen is high, there is also increased CBF and glucose metabolism [81]. Indeed, an increase in choline acetyltransferase (ChAT) activity in the basal forebrain and hippocampus is also observed. For instance, ChAT is responsible for acetylcholine (ACh) synthesis, a neurotransmitter whose level is reduced in AD. Increased ChAT activity reduces the aggregation of A*β* neurotoxicity associated with AD [82, 83].

Although estrogen effects on animal and cellular models are quite beneficial, the impact of reduced estrogen levels due to menopause, oophorectomy, and hormone replacement therapy (HRT) on the risk of AD in women remains controversial. Previous literature reported a reduced risk of AD in women who start HRT within a short period after natural menopause or after oophorectomy [84, 85]. In a cohort study conducted on oophorectomy and aging by the Mayo Clinic, an increased risk (i.e., almost double) of dementia was reported in women who underwent bilateral oophorectomy before menopause [85, 86]. Contrary to it, in women who started HRT just after bilateral oophorectomy and continued with HRT therapy up to the age of natural menopause, there was no risk of developing AD [87].

Contradicting estrogen's beneficial effect, the Women's Health Initiative Memory Study (WHIMS) has shown a reverse effect [88]. In this large randomized clinical trial study of HRT, there was a twofold increase in the risk of dementia among women over 65 years of age. The observational finding could be the result of confounding, which differs from the clinical trial results. Generally, women who use HRT therapy belong to a higher socioeconomic class having a higher education level who can afford better health services. This may be the reason for the lower risk of AD in those women. Another factor could be the timing of HRT therapy [89].

It was proven by observational studies that if initiation of HRT is done around the time when menopause starts (i.e., not too late before menopause), there is a reduced chance of developing AD. Women who started HRT within five years of menopause had a 30% less chance of getting AD as compared to women who never used HRT. However, after five years of menopause, delayed HRT therapy had reverse effects, which did not result in reduced AD risk. On the contrary, they have a double risk of suffering AD, particularly when they started after menopausal age [90].

Other studies, such as the Multi-Institutional Research on Alzheimer Genetic Epidemiology (MIRAGE) and Northern California Kaiser Permanent, also reported a beneficial HRT effect. It starts around menopause with a reduced risk of developing AD, while delayed treatment has reported inverse results with more chances of dementia [87, 91]. The WHIMS trial results were also on a similar track since, in their study, women aged between 65 and 79 years were taken as subjects, and therapy was initiated 10-20 years after menopause.

Currently, two hypotheses are supporting the timely initiation of HRT in women around menopause. The first hypothesis is a window of opportunities. According to this hypothesis, long-term estrogen depletion (LTED) can diminish estrogen receptor-alpha (ER*α*) in the CA1 regions of the hippocampus, which is highly sensitive to estrogen therapy, increasing cognition and neuroprotection [92]. Therefore, estrogen initiation after LTED, when ER*α* receptors are already downregulated, does not result in estrogen's neuroprotective benefit. The other hypothesis is the healthy cell bias of estrogen benefit. It assures that estrogen-only therapy shows its neuroprotective benefit when applied to the healthy neuron, and no beneficial effect was observed on neurons with mitochondrial damage [93].

## 4. AD-Associated Dementia and DM

Perturbed cerebral glucose metabolism, an invariant pathophysiological feature of AD, may play a critical contributor to the pathogenesis of DM [94]. The brain's high energy demand is primarily completed from glucose metabolism, making it vulnerable to impaired energy metabolism. Hence, defective glucose homeostasis heavily affects the brain's cognitive functions, where observations have been documented by many clinical and experimental studies [95]. Substantial research evidence has also shown that in aging animals, performance deficits on a series of cognitive tasks occur due to insufficient cerebral glucose supply, which could be reversed by increasing glucose availability in selective brain areas of aging animals. Microinjection of glucose into the medial septum, hippocampus, striatum, and amygdala can enhance memory processing. These findings indicate that an aging individual is at a greater risk of developing AD due to exposure to glucose deprivation, especially during a highly prolonged cognitive task or training.

Biomarkers may expose the occurrence and severity of hyperglycemia (i.e., diabetes itself) and diabetic vascular complications. In the blood, hemoglobin A1c (HbA1c) may be considered as a biomarker for the presence and severity of hyperglycemia, implying diabetes or prediabetes, or, over time, as a biomarker for a risk factor(i.e., hyperglycemia as a risk factor for diabetic retinopathy, nephropathy, and other vascular diabetic complications) [96].

## 5. Sex Differences in DM

Sex-related changes in lifestyle may cause differences in the risk of developing DM and differences in this disease's occurrence in men and women [97]. A classic example of sex-gender differences is idiopathic diabetes, which accounts for 75% of males' dominance [98]. Remarkably, male predominance starts after adolescence. It is noteworthy that T1DM is characterized by the male : female ratio of 1 : 1 with a slight male predominance [99]. It is expected that around three hundred sixty-six million people will suffer from T2DM worldwide by 2030. Despite the efforts to control it, the number of patients will rise from the current 2.8% to 4.4% of the total human population [100]. The prevalence of T2DM is 10% more in women than in men, similar to the number of women with impaired glucose tolerance, which is 20% more than that of men [101]. The middle-aged population is more affected, surpassing half of the total diabetic subjects, with diabetes occurrence increasing with aging male and female subjects; the highest rates were recorded in older women [102].

It is assumed that sex differences play an imperative role in the pathogenesis of diabetes. Increasing imbalances in sex hormones (such as high progesterone levels in females or testosterone in males) is linked with insulin resistance (IR). Due to the production loss of the endogenous ovarian hormone, women are at higher risk of developing visceral obesity after menopause. Besides, IR triggered oversecretion of the androgen hormone, leading to menstrual disorder in overweight young women. In Japan, for gestational diabetes, diagnostic criteria were revised to properly manage glucose intolerance during pregnancy. Even though glucose intolerance during pregnancy returns to normal after delivery, gestational diabetic patients should be checked regularly for the early detection of T2DM. Besides, practices and behaviors are associated with lifestyles that include nutritional intake and workout, consequently making gender-specific drugs more critical in curing DM [103].

**Figure 3:**
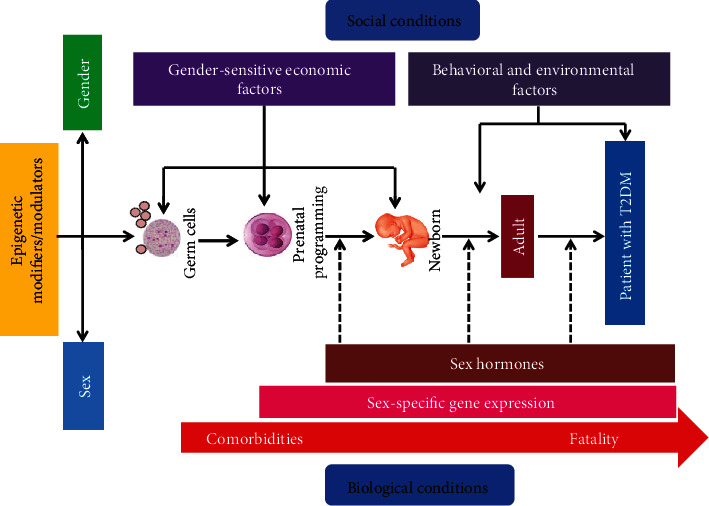
Progression of type 2 diabetes mellitus with social and biological conditions.

T2DM is more commonly diagnosed with age and body mass index (BMI). However, obesity is a considerable risk factor for DM, which is more common in females. Significant differences in the sex ratio are observed across various countries. Striking sex and territorial disparities in the escalation of obesity-related T2DM predominance progressed in the last 30 years, showing a characteristic relationship between dissimilarities in lifestyle, culture, migration, ethnicity, socioeconomic status (SES), social involvement, and gene-environment interactions [104] (Figure 3).

These diversities make differences between males and females in predisposition, development, and clinical presentations. Some more factors, making complications and risks differently in both sexes, are genetic factors, sedentary lifestyle, and epigenetic mechanisms. Lifestyle, environment, and genetic background make a more pandemic increase in T2DM and its related problems.

In T2DM, sex and gender disparities are equally important in its development, presentation, diagnosis, awareness, treatment, and prevention [97, 105]. Females are risk-free up to a broader level of BMI because they accumulate lipids in subcutaneous adipose tissues (SAT), which stimulate less harm than that in visceral adipose tissue (VAT), which takes place in the male [106]. Low levels of testosterone in men are linked with IR and abdominal obesity or central obesity, which are a significant contributor to the development of T2DM in men [107]. DM shows a decrease in the more accommodating favorable group of women's risk factors compared to those of men, causing more considerable differences in abdominal adiposity, where risk factors are associated with coagulation and inflammation, involving DM in normal females than in males [97]. It was observed in a study on Swedish DM patients that male diabetic subjects who were more than 60 years old have extra supportive control of blood pressure (BP) and high blood glucose (hyperglycemia) in comparison to female diabetic subjects, regardless of almost the same medicine given to both sexes. Angiotensin-converting enzyme (ACE) inhibitor was given more frequently to male patients [108].

Another finding suggested that weight reduction of more than three percent of body weight produced a higher decline in DM risk factors in males than in females. Despite the promising effects of intensive lifestyle modification (ILS) in males, baseline risk factors were more abundant in them, likely obscuring any sex differences in incident DM [109]. Pound et al. [110] found that poor diabetic control was noticed in females compared to that in males at all ages from 15 to 17 years (mid teens) onwards. The argument is that it may be due to women's responsibilities with the care of their family and with the management of their DM.

Recently, a study was conducted on German patients to analyze sex differences with adherence and inadequate glycemic control in a group of T2DM subjects. The research outcome exhibited considerable gender-specific dissimilarities with the involvement of adherence and inadequate glycemic control. In males and females, poor glycemic control was noticed in 37% and 19% of the nonadherent participants and in 19% and 18% of the adherent participants [111].

The study of Yoshimura et al. [112] on T2DM in the Japanese population demonstrated that higher energy utilization of each 1000 grams of average body mass is equivalent to the augmentation of the body mass index in males, but not in females. Consumption of soft drinks by men and women and consumption of alcohol, particularly in women, have been correlated with the body mass index's augmentation. From the perception of nutritional intake, these findings specifically indicate that gender differences exist in the pathogenesis of obesity in aged T2DM individuals [112].

Regarding the gestational DM, pregnant women with male fetuses have lesser beta cell activity and higher postprandial glycemic response than pregnant women carrying female fetuses. However, the male fetus is independently linked with increased chances of gestational DM in the mother. It can be concluded that fetal sex is already an unknown factor pertinent to maternal glucose homeostasis in pregnancy [113]. Siddiqui et al. [114] reviewed that male diabetic patients live more effectively with DM by displaying lesser dismay, by having worries but remaining lively, and by keeping a positive attitude. They manage their illness more optimistically and undergo minor social stresses. Sex differences become more important when one must accept to survive effectively with diabetes. Diabetic women wish to build up a positive attitude towards the disease and its management [114].

An examination of the types and treatments of DM highlights three key factors such as (a) it is a long-lasting and mentally and interactively challenging disease, (b) it is impossible to get rid of this disease, and (c) diabetes may be managed but cannot be cured. Diabetic individuals must learn to survive with this problem [114].

More studies are needed to unveil the process of how T2DM is pathophysiology linked with sex-dimorphic and diabetes-related problems that could help to discover ways for a more personalized care in the future and to encourage further awareness regarding sex and gender-specific risk factors.

## 6. Pathophysiological Mechanisms of AD and T2DM

**Figure 4:**
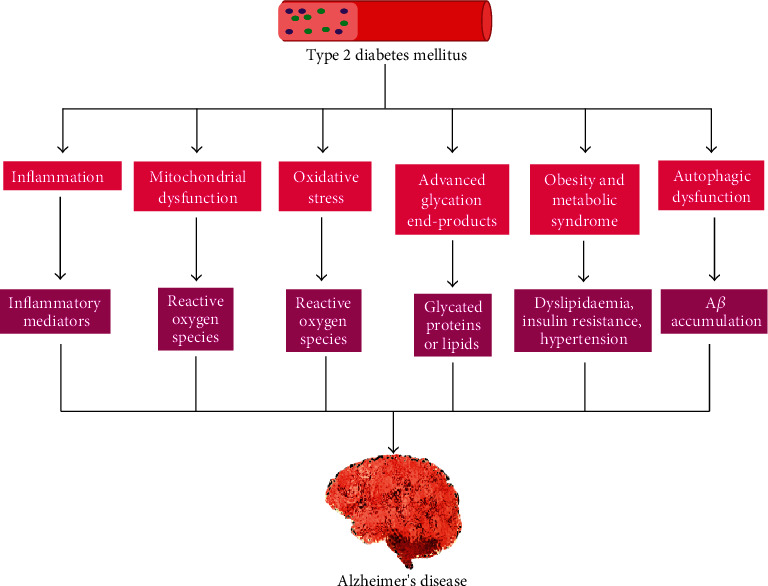
The outline of pathogenic mechanisms by which type 2 diabetes mellitus can cause Alzheimer's disease pathogenesis.

In the case of both AD and T2DM, it has been found that there was a noticeable impairment of energy and glucose metabolism, which has been revealed by magnetic resonance imaging (MRI) and positron emission tomographic (PET) studies [27]. In both these diseases, amyloid genesis remains a principal feature. Similarly, in the pancreatic islets of Langerhans in T2DM patients, islet amyloid polypeptide (IAPP) deposits were identified [115]. Intriguingly, fibrils and oligomers with more severe diabetic traits similar to AD mouse models that overexpress APP have been developed from diabetic mice overexpressing IAPP [116]. Under conditions of oxidative stress and endoplasmic reticulum stress, advanced glycation end products (AGEs) and their receptors (RAGE) are gathered in the sites of diabetic complications, including atherosclerotic plaques, retina, and kidney [117]. Likewise, tau and glycated products of A*β* form in transgenic AD models and postmortem brains of AD individuals under similar conditions of stress forming a vital component of NFTs [118]. Additionally, in T2DM and AD cases, there were numerous mutual pathophysiological features [119], as shown in Figure 4.

### 6.1. Inflammation

In T2DM, IR is regarded as an essential feature, often accompanied by inflammation, especially with raised levels of the inflammatory mediators like *α*-1-antichymotrypsin, C-reactive protein (CRP), and interleukin-6 (IL-6). Furthermore, it is hypothesized that raised levels of products of the acute-phase reaction in inflammation are associated with immunological dysfunction, leading to IR. Similarly, there is proof that inflammatory processes are related to AD [120, 121]. On the other hand, in AD patients, inflammatory products gather at different rates compared to healthy control subjects. Moreover, in AD patients, IL-6 is present in senile plaques [122], and raised immunoreactivity to IL-6 is noticed in ventricular and lumbar cerebrospinal fluid.

Few studies have associated CRP with a raised risk of AD [123, 124]. Interestingly, some studies support the fact that there is a reduced incidence of AD in individuals who are prescribed nonsteroidal anti-inflammatory drugs (NSAIDs) to treat chronic pain [125, 126]. Remarkably, peroxisome proliferator-activated receptor-*γ* (PPAR*γ*) agonists have been found to have anti-inflammatory effects [127].

### 6.2. Mitochondria and Oxidative Stress

In the pathogenesis of both T2DM and AD, oxidative stress and mitochondrial dysfunction have a crucial contribution, signifying a possible association [128]. It has also been observed that, in T2DM, there is a raised level of oxidative stress along with a lower level of antioxidant capacity [128], which suggests that this can ultimately lead to neuronal injury with mitochondria as specific targets [129]. Conversely, when studied in a rat model of T2DM, it has been found that brain mitochondria exhibit age-related weakening of the respiratory chain and uncoupling of oxidative phosphorylation [130], which is essential for the production of adenosine triphosphate (ATP).

Because the mitochondria provide approximately 90% of the ATP required for normal neural functions, mitochondrial dysfunction can cause loss of metabolic control and neural degeneration. Since the brain is profoundly reliant on ATP production, it is more vulnerable [131]. Besides, as per the mitochondrial cascade hypothesis, the rate of mitochondrial damage accumulation is determined through the basal rate of production of reactive oxygen species (ROS) by the electron transport chain, which in turn is determined by genetics. Thus, oxidative changes in mitochondrial proteins, lipids, and nucleic acids increase ROS production and stimulate the cells towards A*β* production, NFTs formation, and tau phosphorylation [132].

### 6.3. Advanced Glycation End Products

Advanced glycation end products (AGEs) are produced when reducing sugars do not enzymatically react with the amino groups of proteins and then go through further reactions (including condensation, dehydration, and rearrangement) to turn into irreversibly cross-linked heterogeneous derivatives [133]. Interestingly, in 1912, AGEs were initially identified as Maillard reaction's end products [134]. Although AGEs can build up in several cells because of normal aging, the accumulation rate is markedly increased in DM [135].

Interestingly, in AD, the formation of an elevated level of AGEs is also noticed. Nonetheless, in AD, extracellular accumulation of AGEs is more likely to be produced by the enhanced oxidation of glycated proteins, such as redox-active iron bound to proteins in amyloid plaques [136]. Alternatively, in both DM and AD, intracellular accumulation of AGEs is generated through the presence of phosphates and reactive sugars, for instance, fructose. The metabolic consequences include hypometabolism of glucose, impaired cell function, and oxidative stress [137]. AGEs have also been observed in the central nervous system of diabetic patients, which could offer a mechanistic connection [96].

### 6.4. Obesity and Metabolic Syndrome

Obesity, particularly central body obesity, is regarded as an independent risk factor for metabolic syndrome, a disorder of hypertension, IR, and dyslipidemia. Furthermore, for the development of T2DM, metabolic syndrome and obesity are vital risk factors [138, 139]. The evidence found in the following studies indicates that there might be a connection with AD as well. In the Baltimore Longitudinal Study of Aging, a raised AD occurrence rate was reported among females with a BMI greater than 30 and males between 30 and 45 years old with weight gain [140, 141].

Similarly, another study found that males and females with a midlife BMI greater than 30 have an increased risk for AD [142]. On the other hand, a Swedish study found that with every 1.0 increase in body mass index at the age of 70, the risk of AD increased by thirty-six percent [143]. In another study, it was found that individuals with AD have a considerably lower mean plasma concentration of high-density lipoprotein cholesterol, higher mean plasma concentration of glucose and triglycerides, and larger mean waist circumference [144]. In terms of the regulation of brain functions, a significant contribution of leptin may also be present. In recent times, it has been recommended by Han and Li [145] that research on the suggested connection between AD and T2DM would progress by studying the faulty signaling of leptin in connection with the lack of a disturbed signaling of insulin [145].

### 6.5. Autophagic Impairments in AD and T2DM

In most neurodegenerative diseases, the intracellular buildup of misfolded protein aggregates is a significant characteristic [146]. The aforesaid protein aggregates are found to be cleared from the neurons by the process called autophagy. Autophagy is also significant for the maintenance of neuronal homeostasis [147, 148]. As neurons age, they accrue harmful intracellular protein aggregates and damaged organelles, including mitochondria, that must be straightaway cleared for the neurons' proper functioning at a physiological level [149].

Recently, experiments have revealed that autophagic machinery controls the normal function of pancreatic *β*-cells, and it is also associated with the T2DM pathophysiology. IR leads to the generation of oxidative stress on insulin-responsive tissues. In the previously mentioned cases, an elevated level of autophagy plays a protective role [150]. Besides, except for indirect effects, some researchers describe the direct influence of IR on autophagy by inhibiting the mechanistic target of rapamycin (mTOR) signaling pathway [151].

## 7. Mechanistic Linkage of T2DM and AD Hallmarks

**Figure 5:**
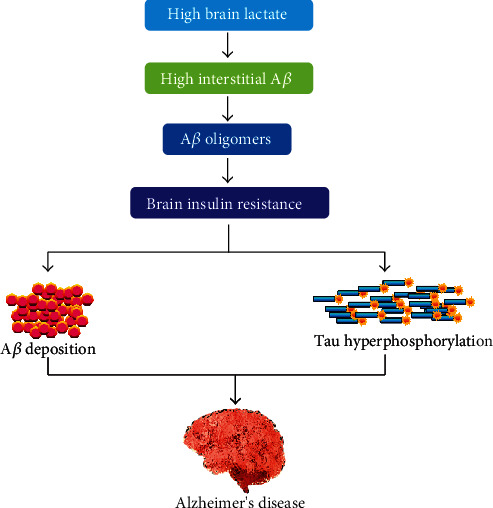
The role of insulin resistance in the pathogenesis of Alzheimer's disease.

Evidence suggested that brain IR intensely stimulates the formation of A*β* and tau pathologies [152–154], as stated in Figure 5.

### 7.1. A*β* Pathology

It has been indicated by growing research that hyperinsulinemia may confer the risk of AD by regulating the toxicity of A*β*. The insulin-degrading enzyme is responsible for the degradation of the A*β* protein; moreover, it degrades insulin [116]. On the other hand, in T2DM, insulin concentration, which plays a role as a competitive substrate for the insulin-degrading enzyme, is increased via peripheral hyperinsulinemia.

In contrast, peripheral hyperinsulinemia can inhibit the A*β* degradation that progressively builds up to produce insoluble plaques. In both glia and neurons, the insulin-degrading enzyme has formerly been recognized as the primary A*β* regulator [155]. Interestingly, in Tg2576 AD transgenic mouse models, by investigating the influence of IR induced by diet on amyloidosis, Ho et al. [156] observed that only at six months of age do the animals exhibit the first signs of memory deficits. The aforesaid research was consistent with the theory that, for the detected elevated comparative risk for neuropathology of AD, IR might play an essential role and reveals the first proof to recommend that IR signaling can affect the production of A*β* in the brain.

In people affected with T2DM, increased plasma glucose levels are regarded as a commonly observed pathological characteristic. A convincing association between AD and glucose metabolism was established in a study that stated that hyperglycemia could modulate the extracellular A*β* concentrations and neuronal activity *in vivo*. All these findings collectively signify that the activation of the ATP-sensitive potassium channel can mediate the response of hippocampal neurons towards hyperglycemia through coupling metabolism with neuronal action and brain interstitial fluid levels of A*β* [157].

In the case of lipid metabolism, insulin has been found to have a significant contribution. Moreover, increased free fatty acid synthesis and elevated lipolysis are caused by the weakening of insulin signaling [122]. In the plasma membrane, the interaction among APP and cholesterol is essential for the synthesis and clearance of A*β*, as revealed by recent studies. Captivatingly, elevated A*β* production was exhibited by the Tg2576 AD mice raised on a diet containing high cholesterol and high fat [158].

### 7.2. Tau Pathology

A host of phosphatases and kinases regulates a complex balance between dephosphorylation and tau phosphorylation to preserve neuronal homeostasis under physiological conditions [159]. While components of the mitogen-activated protein kinase (MAPK) pathway lie downstream of one arm, glycogen synthase kinase-3-beta (GSK-3*β*) and protein kinase B (PKB or AKT) are found to be located downstream at the other arm of the insulin signaling pathway [160]. Again, AKT keeps GSK-3*β* inactive and at the inhibitory serine-9 residue, which phosphorylates GSK-3*β*. Through phosphorylation at the tyrosine-216 residue, GSK-3*β* is converted to its active form under conditions of IR.

In the AD brains, active GSK-3*β* causes hyperphosphorylation of tau to produce pathological epitopes, namely, PHF1, AT8, and AT100, which make up NFTs and pretangles [161]. Phosphatases, particularly protein phosphatase 2A (PP2A), are found to regulate tau. Furthermore, at a physiological level, PP2A can cause dephosphorylation of kinases p70S6K and GSK-3*β* to preserve phosphorylation of tau [162]. Fascinatingly, some investigators have revealed PP2A downregulation in both T2DM and T1DM mice. The aforesaid finding recommends that IR might exacerbate the phosphorylation of tau via the downregulation of PP2A [163, 164]. On the other hand, apoptosis is stimulated by diabetes through the caspase-3 activation in affected tissues [165]. In a different study, Kim et al. [166] demonstrated an elevated level of tau cleavage and phosphorylation in db/db mice's brains through an animal model of T2DM for diabetic dyslipidemia.

## 8. Emerging Therapeutic Approaches in AD and Diabetes

Despite the efforts to develop therapies for AD, success at the clinical level is still unsatisfactory. Several strategies for managing AD, such as immunotherapy against A*β*, antioxidants, anti-inflammatory agents, natural products, and nutrition-based approaches, have been tried. Presently, for the treatment of mild to moderate AD, cholinesterase inhibitors (i.e., donepezil, rivastigmine, and galantamine) are used, whereas the N-methyl-D-aspartate (NMDA) receptor antagonist (i.e., memantine) is used for the treatment of severe cases of AD [167]. The major problem with using these drugs lies in providing symptomatic and short-term benefits only without affecting the pathogenic mechanisms involved in the disease [168]. Rigorous work has been done in drug discovery in the last decade to develop disease-modifying drugs to counter AD progression. Because of the slow progression of the pathophysiological process in AD, it is necessary to develop such drugs, which target this disease at the early presymptomatic stage, when the disease is still hidden.

### 8.1. Insulin

Insulin, which is primarily secreted by the pancreas' *β*-cells, regulates blood glucose levels [169]. Insulin, through receptors located in the olfactory bulb and thalamus, executes several brain functions, like food intake and cognitive function, including memory [170]. Impairment in regulating blood glucose levels and IR may be linked to ACh synthesis, which is linked to the neurodegenerative disorder in diabetes, by the ACh transferase enzyme, which is expressed in insulin receptor-positive cortical neurons [171]. In individuals with early MCI and AD, verbal memory recall, especially in APOE4-individuals, was facilitated due to acute intranasal administration of 40 or 20 IU of insulin. However, following administration of insulin, memory-impaired *APOE4* individuals exhibited lesser recall, suggesting the role of the mediating effects of insulin in CNS [172].

Further experiments with the same group have confirmed that three weeks of intranasal insulin enhanced functional status, verbal memory, and attention in MCI and AD individuals. This treatment has elevated plasma concentrations of the short form of A*β* peptide, resulting in an enhanced ratio of A*β* 40/42 [173]. The actions of insulin on cognition are dose dependent (with maximal effect at 20 IU) and controlled by the genotype of *APOE* [174].

### 8.2. Insulin Secretagogues

Glimepiride binds with sulfonylurea receptor SUR1, which is present on the pancreatic cell's membrane, and this binding can eventually induce secretion of insulin by closing the potassium channel [175]. Glimepiride is also found to have additional pancreatic actions, including activating PPAR*γ*, inducing the release of glycosylphosphatidylinositol- (GPI-) anchored proteins, and elevating glucose uptake [176]. It has been reported that it docks to PPAR*γ* and shows PPAR*γ* agonistic activity in a cell-based transactivation assay [177]. Besides, it can upregulate the PPAR*γ* target gene expression, including leptin and aP2, also increasing the interaction of PPAR*γ* with cofactors [178]. On the other hand, it has been revealed that the activation of PPAR*γ* can reduce senile plaque levels and A*β* levels, which, in turn, improve the cognitive function in AD individuals [179].

### 8.3. Insulin Sensitizers

Metformin is most commonly used as an orally active biguanide. It can reduce insulin resistance through potentiating insulin action and lowering glucose effects (i.e., by subduing gluconeogenesis in the liver). Moreover, through AMP-activated protein kinase (AMPK), metformin can enhance the insulin sensitivity of skeletal muscle and liver [180]. In a study by Gupta et al. [181], the authors stated that upon exposure to metformin, increased insulin effects could prevent AD-related pathological and molecular features in a cell culture model of insulin resistance. On the other hand, following treatment with metformin, enhanced viability of neurons was observed in an *in vitro* model of ischemia [182]. Henceforth, in decreasing neuronal cell injury and neuropathy linked with hyperglycemia in diabetes, the use of metformin might be useful. In human subjects, it was observed that treatment with metformin markedly reduces the risk of dementia, as revealed by a Taiwanese clinical study [183]. Some T2DM medications and their impact on AD are shown in Table 1.

### 8.4. Amylin Analog

Amylin is a small peptide hormone, which is secreted along with insulin from the pancreatic *β*-cells. Interestingly, amylin shares several similar features with A*β*, including being degraded by an insulin-degrading enzyme and having a similar *β*-sheet structure [184]. BBB can also be crossed by amylin and appears to contribute to the regulation of anxiety, mood, and memory [185]. Due to its amyloidogenic potential, the Food and Drug Administration has approved its analog pramlintide for T1DM and T2DM. It has been found that plasma levels of amylin are markedly decreased in AD individuals. Reduced neuroinflammation, decreased oxidative stress, and improved memory might be observed due to pramlintide administration, as revealed by preclinical results in AD mouse models [186]. Further studies are required to evaluate the potential role of amylin and its analog in AD.

### 8.5. PPAR*γ* Agonists

In AD patients' brains, PPAR*γ* found in a significant amount is considered a critical neuromodulator [187]. PPAR*γ* contributes to many processes involved in the pathogenesis of both AD and diabetes, including cell differentiation and growth and metabolic and inflammatory processes [188]. Originally, thiazolidinediones (i.e., pioglitazone and rosiglitazone) were used to explore the contribution of PPAR*γ*. Their mechanism involves stimulation of PPAR*γ* activity in response to insulin alterations, thus causing a drop in the serum glucose level [189]. In addition, these drugs promote neuronal Ca^2+^ homeostasis in the hippocampus [190], improve IR [191], promote cholesterol homeostasis [192], and decrease cerebral inflammation via inhibition of tumor necrosis factor and interleukin-6 [193]. Such activities are postulated to improve AD patients' cognitive function and regulate A*β* peptide proliferation [194]; thus, it can prevent many dementia cases in upcoming years by controlling these risk factors [195].

## 9. Conclusion and Future Perspectives

The prevalence of AD and other types of dementia across most regions of the world is higher in women than in men, especially in the elderly. The sex differences in the brain begin with sex-determining genes and fetal hormonal programming. Understanding these differences can significantly impact risk estimation, monitoring, and management of brain disorders. A cohort study published in 2015 showed that women who are positive for the *APOE4* are more prone to develop AD than men. Instead of pooling data for sexes, more effort is needed to identify numerous other factors implicated in dementia by sex, speeding up efforts to explore new directions for personalized treatment and managing various dementia types. Regarding AD, studies indicate that there is a more rapid progression of hippocampal atrophy in women than in men. Nevertheless, men progress towards AD, probably due to the severe periventricular white matter hypersensitivities and reduced global cognitive performance. In terms of clinical presentation, men show more aggressive behavior, more comorbidity, and more mortality, while women have more affective symptoms and disabilities with longer survival time.

Besides genetic and brain-based cognitive risk factors, social factors also affect the cause, risk, and outcome of different dementias. Educational and occupational levels are two major social causes, which affect both genders, and some social stigma is associated with these factors, particularly in the older generation. Behavioral and lifestyle factors such as physical exercise, diet, nutrition, smoking, and alcohol abuse affect both genders differently, thereby influencing different dementia types, particularly vascular dementia. The number of diabetic patients with neurological manifestations has been continuously increasing, with many epidemiological studies reporting an intricate linkage between diabetes and AD occurrence. T2DM and AD share many mutual pathophysiological features like glucose metabolism and noticeable impairment of energy. Oxidative stress, accumulation of intracellular AGEs, and mitochondrial dysfunction have a crucial contribution in both these diseases, signifying a possible association. Insulin resistance and leptin signaling are turning out to be potential target mechanisms involved in diabetes-induced AD pathology. The global economic burden of AD-dementia care and cure is enormous. Hence, there is an immediate need for extensive sex-based studies to assess AD-associated dementia and therapeutic strategies.

## Figures and Tables

**Table 1:** Type 2 diabetes medications and their impact on AD.

Classification	Mechanism	Examples	Impact on AD	References
Biguanide (insulin sensitizers)	(i) Primarily decreases hepatic glucose production (ii) Increases muscle glucose uptake	Metformin Metformin ER Metformin solution	(i) Increases *β* secretase level (ii) Decreases tau phosphorylation (iii) Prevents acetylcholine esterase activity	[196–199]

Thiazolidinedione (insulin sensitizer)	(i) Selective PPAR*γ* antagonist (ii) Increases glucose transport into adipose, muscle, and liver cells	Rosiglitazone Pioglitazone	(i) Reduces A*β* levels (ii) Decreases cerebral glucose utilization and increases ROS production (iii) Prevents expression of IL-6, TNF-*α*, and COX-2	[198–201]

Glucosidase inhibitor	(i) Interferes with alpha-glucosidase, thereby inhibiting the hydrolysis and absorption of carbohydrates in the GI tract	Acarbose Miglitol	(i) Decreases oxidative stress (ii) Prevents brain aging and improves cognition (iii) Reduces insulin resistance	[198, 199, 202, 203]

Sulfonylurea (insulin secretagogue)	(i) Enhances insulin secretion by their interaction with ATP-sensitive K channel on the beta cell membrane	Glimepiride Glipizide Glyburide	(i) Decreases tau phosphorylation (ii) Decreases lipid peroxidation (iii) Improves cognition	[204, 205]

Meglitinide	(i) Blocks ATP-dependent potassium channels (ii) Stimulates insulin release from the pancreatic beta cells	Repaglinide Nateglinide	(i) Protects against dementia and improves cognition	[198, 199, 206, 207]

Amylin analog	(i) Slows gastric emptying, promotes satiety, and suppresses the abnormal postprandial rise of glucagon	Pramlintide acetate	(i) Neuroprotective effects	[186, 208]

GLP-1 analog	(i) Dose-dependent and glucose-dependent augmentation of insulin secretion (ii) Reduces gastric emptying time, suppresses inappropriately elevated glucagon levels, and leads to weight loss	Exenatide Exenatide ER injection	(i) Reduces APP and A*β* levels (ii) Increases neurogenesis and cognition	[198, 199, 209, 210]

DPP-4 inhibitor	(i) Prolongs active incretin levels (ii) Increases insulin synthesis and release from pancreatic beta cells and decreases glucagon secretion from pancreatic alpha cells	Saxagliptin Linagliptin Sitagliptin	(i) Reduces APP and A*β* deposits (ii) Decreases tau phosphorylation (iii) Improves cognition	[211–213]
